# From classical approaches to new developments in genetic engineering of live attenuated vaccine against cutaneous leishmaniasis: potential and immunization

**DOI:** 10.3389/fpubh.2024.1382996

**Published:** 2024-07-05

**Authors:** Zahra Rooholamini, Hassan Dianat-Moghadam, Mahsa Esmaeilifallah, Hossein Khanahmad

**Affiliations:** ^1^Department of Genetics and Molecular Biology, School of Medicine, Isfahan University of Medical Sciences, Isfahan, Iran; ^2^Pediatric Inherited Diseases Research Center, Isfahan University of Medical Sciences, Isfahan, Iran; ^3^Department of Parasitology and Mycology, School of Medicine, Isfahan University of Medical Sciences, Isfahan, Iran

**Keywords:** attenuated vaccines, CRISPR, cutaneous leishmaniasis, drug resistance, leishmanization, immunization

## Abstract

Despite the development of a vaccine against cutaneous leishmaniasis in preclinical and clinical studies, we still do not have a safe and effective vaccine for human use. Given this situation, the search for a new prophylactic alternative to control leishmaniasis should be a global priority. A first-generation vaccine strategy—leishmanization, in which live *Leishmania major* parasites are inoculated into the skin to protect against reinfection, is taking advantage of this situation. Live attenuated *Leishmania* vaccine candidates are promising alternatives due to their robust protective immune responses. Importantly, they do not cause disease and could provide long-term protection following challenges with a virulent strain. In addition to physical and chemical methods, genetic tools, including the Cre-*loxP* system, have enabled the selection of safer null mutant live attenuated *Leishmania* parasites obtained by gene disruption. This was followed by the discovery and introduction of CRISPR/Cas-based gene editing tools, which can be easily and precisely used to modify genes. Here, we briefly review the immunopathology of *L. major* parasites and then present the classical methods and their limitations for the production of live attenuated vaccines. We then discuss the potential of current genetic engineering tools to generate live attenuated vaccine strains by targeting key genes involved in *L. major* pathogenesis and then discuss their discovery and implications for immune responses to control leishmaniasis.

## Graphical Abstract

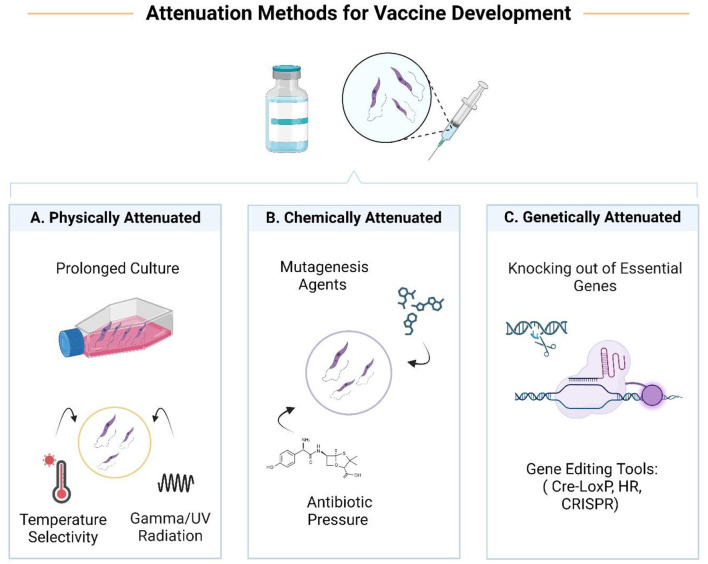

## Introduction

**Figure 1:**
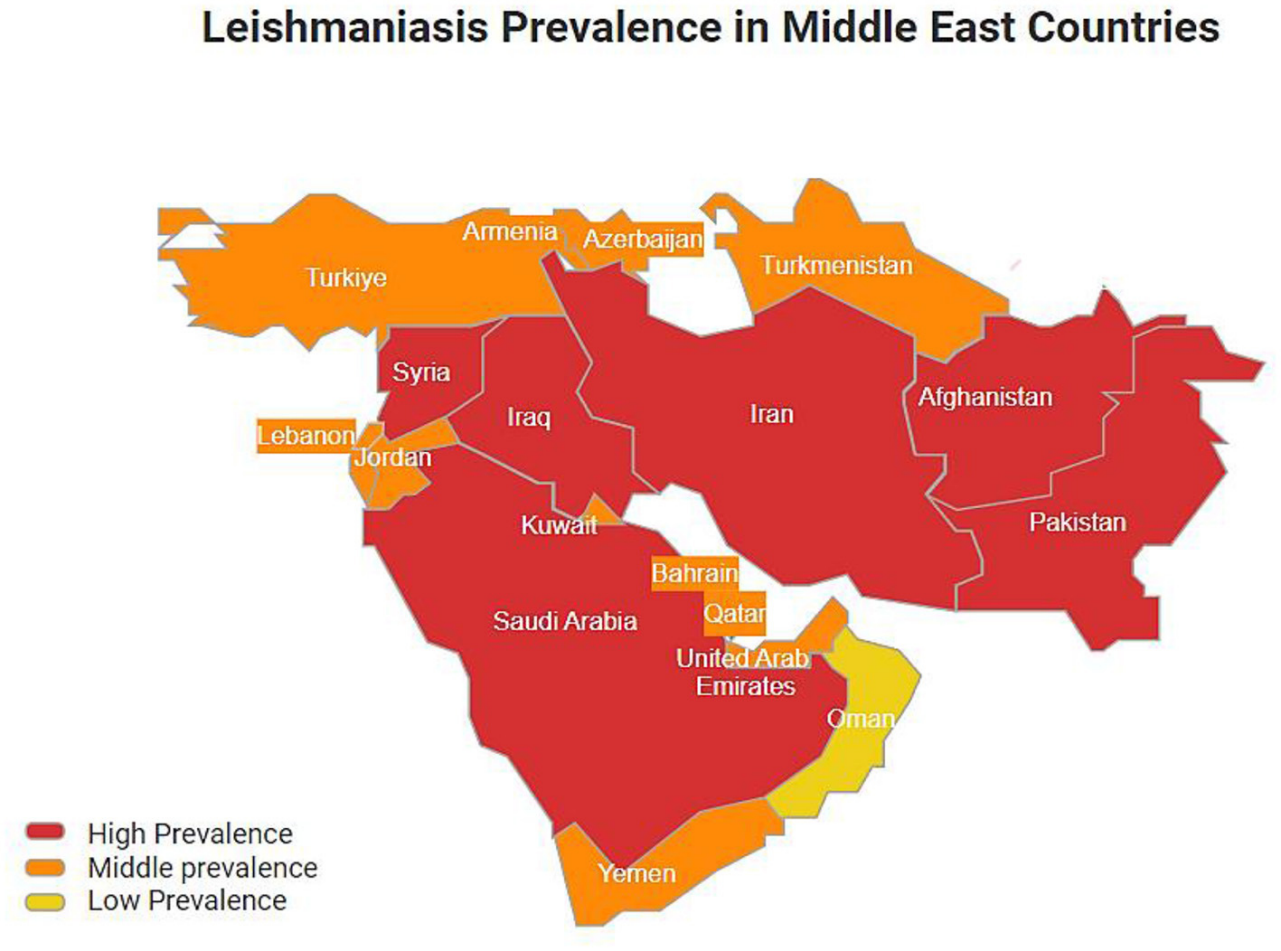
Leishmaniasis prevalence in different Middle East countries.

Leishmaniasis is a vector-borne infection caused by *Leishmania*—an obligate intracellular protozoan parasite. The two morphologically distinct forms of this are the promastigote, which is passed on by female Phlebotomine sandflies, and the amastigote, which occurs in mammalian hosts. The several clinical forms of the disease can be grouped into three main clinical forms: visceral (VL), mucocutaneous (MCL), and cutaneous leishmaniasis (CL) (1). CL is a painless and chronic ulcer at the site of sandfly bites and is the most common clinical syndrome in many affected regions, especially in the Middle East, where it has been reported in two main forms: zoonotic CL (ZCL) caused by *Leishmania major* and anthroponotic CL (ACL) caused by *Leishmania tropica* and mixed infection with them, which is high there (2, 3). In 2022, WHO reported that 85% of the global CL incidence occurred in eight countries, Afghanistan, Iran, Iraq, Syria, Algeria, Brazil, Colombia, and Peru (4). New outbreaks in the Middle East in recent years have been linked to wars in Syria, Yemen, Turkey, and Iraq. Refugee migration from endemic to non-endemic areas and vice versa, poor hygiene, malnutrition, weak immune systems, poor housing, lack of resources, environmental conditions, climate change, poor urbanization management, use of agricultural lands for residential purposes, and changes in vector populations link to a substantial rise in CL prevalence, which are present circumstances in most of the Middle East (3, 5, 6) (Figure 1). Although the first line of treatment of leishmaniasis with pentavalent antimonials is affordable and generally available in many endemic countries in the Middle East, economic sanctions, war, and counterfeit drug markets make access to the standard treatment difficult. In addition, the efficacy of this type of treatment is variable due to drug resistance and induction of organ toxicity (2, 3).

Fortunately, the development of immunity to the parasite in infected individuals following rehabilitation has highlighted the role of vaccination in disease management (5). In addition, the partial understanding of the immunopathogenesis of leishmaniasis has motivated immunologists and researchers in the leishmaniasis field to investigate and develop the different types of vaccines required. In the early 20th century, controlled inoculation of live virulent *L. major* promastigotes was used to immunize people in hyperendemic regions, preventing parasite infection in up to 80% of people. However, leishmanization as effectively powerful to control CL had several disadvantages that led to its abandonment (except in Uzbekistan, where this method is still used), including permanent skin lesions, safety concerns about HIV transmission, limitations in immunosuppressed people, and issues with Good Manufacturing Practice (GMP) standards (7, 8). Given these challenges, vaccine development shifted to inactivated vaccines. Due to the simplicity and cost-effectiveness of the production process, inactivated vaccines have been developed in various formulations. They are considered safe human vaccines and have been used as an alternative medication for drug-resistant type CL (9). Inactivation of the parasite while preserving the antigenic structures has been achieved by physical methods such as heat, chemicals, sonication, or UV radiation. This category has been studied in many clinical trials, but none of them have been approved by the World Health Organization (WHO) due to the lack of remarkable efficacy and the need for multiple vaccine doses (7, 8).

In addition to extended vaccines based on whole organism components, purified immunogenic fragments of the parasite have been developed as vaccine candidates, reducing the possibility of adverse reactions. Leishmune^®^–a commercial canine vaccine consisting of fucose–mannose ligand (FML) from *Leishmania donovani* adjuvanted with QuilA saponin shows moderate clinical signs and lesions in vaccinated/infected dogs (8, 10). Remarkable advances in molecular biology have led to a new generation emergence of subunit or synthetic leishmaniasis vaccines based on membrane or soluble parasite proteins, replacing the previous native form vaccines. Cost-effectiveness and a straightforward manufacturing process allow their large-scale production. There is no live pathogen and no risk of infection in immunosuppressed individuals. With all these advantages, there are also some disadvantages, including an attempt to escape immune system deactivation and increased immunogenicity. Variations in the final conformation and structure of peptides occur due to heterologous expression systems, which could almost be related to post-translational modifications. Also, the epitopes could be selected to induce the desired immune response, and a particular antigenic arrangement could be chosen to induce a milder immune response (8, 11).

The *Leish*-111f vaccine is a tandem combination of three highly conserved *Leishmania* antigens, thiol-specific antioxidant (TSA), *L. major* stress-inducible protein 1 (LmSTI1), and *Leishmania* elongation initiation factor (LeIF), resulting in a 111 kDa polyprotein. In addition, studies indicated that *Leish*-111f formulated in IL-12 induces antibody response and IFN-γ production as well as soluble *Leishmania* antigen (SLA), but MPL-SE is considered a suitable alternative due to problems related to the manufacturing process and uncertainty of safety (12). *Leish*-111f is the first leishmaniasis vaccine to demonstrate immunogenicity in human clinical trials (12). In human clinical trials, *Leish*-111f is the first leishmaniasis vaccine that has demonstrated immunogenicity. A total of 77 healthy Indian subjects, with or without previous exposure to *Leishmania*, were administered three doses of *Leish*-F1+MPL-SE and followed for 168 days. Results showed safe and mild reactions associated with an increase in Th1-type cytokines (13). Purified peptides from different hosts administered with CpG adjuvant in BALB/c mice and eukaryotically expressed vaccine resulted in greater immune protection than the prokaryotic vaccine due to critical modifications that occur during protein construction in *L. tarentolae*, such as glycosylation, which involves the attachment of carbohydrate molecules to the N- or C-terminus of proteins, responsible for efficient peptide folding and interaction. Moreover, many studies have shown that glycosylation improves the immunogenicity and duration of conjugated vaccines compared to non-glycosylated vaccines (14, 15). Recently, significant advances in gene editing tools and *Leishmania* genome manipulation and generation of mutant weakened parasites have been explored as a desirable means of disease management. In this paper, we have reviewed the development of genetically live attenuated *Leishmania* vaccines.

## *Leishmania* immunology

Following the entry of *Leishmania* promastigotes into the host's dermal layer via the sandfly bite, the parasites reside in phagocytic cells such as tissue macrophages and dendritic cells or neutrophils. *Leishmania* GP63 directly uses complement C3 cleavage to prevent complement-mediated lysis, allowing C3bi to interact with the phagocytic cell receptor CR3 for facilitating attachment and uptake. Activated dendritic cells migrate from sites of antigen acquisition to draining lymph nodes and present *Leishmania* antigens to naive T cells, accompanied by the production of cytokines leading to CD4+ and CD8+ activation. The future fate of the parasite depends on the polarization and the final phenotype of the macrophages. The differentiation of macrophages into pro-inflammatory (M1) or anti-inflammatory (M2) phenotypes, known as macrophage polarization, plays a critical role in the immune response to leishmaniasis. Resistance to *Leishmania* infection is associated with the M1 phenotype, whereas the M2 phenotype dominates in susceptible environments. The balance between M1 and M2 macrophage polarization can be regulated by cytokines produced by CD4+ Th1 and Th2 lymphocyte subpopulations. The M1 macrophage polarization is mainly due to LPS, IFN, TNF, and GM-CSF, which also activates the complement system and recruits the immune cells. The polarization of macrophages into the M2 subset by the *Leishmania* parasites under secretion of Th2 cytokines and reduction of dendritic cells results in a decrease in antigen presentation and an immunosuppressive environment that supports their survival (16–18). Toll-like receptors (TLRs) play a key role in enhancing the immune response in the context of cutaneous vaccination by identifying pathogens. Some TLR ligands, such as prokaryotic CpG oligodeoxynucleotide (ODN) motifs, are considered effective adjuvants identified by TLR9. CpG ODNs induce the production of pro-inflammatory cytokines, including IL-12 and IFN-γ, which promote the development of a Th1 immune response (19). In a case-control study, gene expression measurement of IL-12 P40, IFN-γ, IL-1β, IL-4, and IL-10 from peripheral blood mononuclear cells (PBMC) of patients with anthroponotic cutaneous leishmaniasis (ACL) who responded and those who did not respond to meglumine antimoniate treatment showed a significant increase in Th1 cytokines (IL-12 P40, IFN-γ, and IL-1β) in the responsive group and Th2 cytokines (IL-4 and IL-10) in the non-responsive group (20). It has also been reported that the CD4+ T-cell response weakens in people with symptomatic visceral leishmaniasis but could return along with central memory T-cells that induce immunity after medication (21).

## Strategies to produce attenuated vaccines

Attenuated vaccines could be produced by limiting the pathogenicity of the parasite through some techniques (Table 1). Weakened pathogens as whole-organism vaccines could present a set of antigens to the immune system, limiting the effect of antigenic polymorphism and genetic variation (22). It could also simulate actual infection and potentially activate the Th1 immune response. But sometimes, depending on the attenuation method, important immunogenic epitopes cannot be generated. This is a major drawback that limits the use of attenuated vaccines in immunosuppressive conditions such as HIV infection, organ transplantation, chemotherapy, or pregnancy. Strategies used to attenuate parasites based on defined and undefined genetic alterations include chemical, physical, and genetic attenuation (Table 1).

**Table 1:** Different live attenuated leishmaniasis vaccines according to attenuation approach.

**Attenuation method**	**Species**	**Animal model**	**Result**	**References**
**Physical approaches**				
Prolonged *in vitro* culture	• *Leishmania major* • *Leishmania tropica*	C57BL/6, BALB/c.H-2^b^, BALB/c.H-2^k^, BALB/c	BALB/c, BALB/c.H-2^b^, and BALB/c.H-2^k^ have been protected partially against CL	(27)
Prolonged *in vitro* culture	*Leishmania chagasi*	BALB/c	Without immunization	(28)
Prolonged *in vitro* culture	*Leishmania amazonensis*	C57BL/6	Decrease in parasite burden and increase in IFN-γ amounts	(29)
Temperature selectivity and treatment with mutagenesis agent	*Leishmania braziliensis*	BALB/c	Complete protection against infection and reduced in lesion size	(30)
Gamma irradiation	*L. major*	CBA, BALB/c	High protection after subcutaneous challenge with *L. major*	(31)
**Chemical approaches**				
Chemical mutagenesis (N-methyl-N-nitro-N-nitrosoguanidine)	*L. major*	BALB/c	Reduced lesion size	(32)
Gentamicin pressure	• *L. major* • *Leishmania mexicana* • *Leishmania infantum* • *Leishmania donovani*	BALB/C	Induced protection and no skin lesion	(33)
Gentamicin pressure	*L. infantum*	Dogs	No clinical manifestation and parasite in internal organs, higher IFN-γ	(34)

Physical methods include techniques such as prolonged subculture, use of radiation (gamma rays or UV), and temperature sensitivity. Treatment with mutagenic agents or promastigote culture under antibiotic pressure is considered chemical attenuation. The gentamicin-attenuated *L. major* vaccine is now in clinical trials and has shown promising results in mice and humans.

On the other hand, it also defined modifications that lead to the knocking out of genes responsible for pathogenicity. Today, this approach could be a suitable alternative that reduces the potential for reversibility (23–25). In addition, unlike the old method of leishmanization, mutant parasites altered using precise gene manipulation tools led to the appearance of an improved leishmanization in terms of non-pathogenicity and protection against all divergent *Leishmania* species (26).

## Genetically attenuated parasites

### Good candidate gene for attenuated vaccines

Live attenuated *Leishmania* vaccines as non-pathogenic parasites that provide the immune system with whole antigens that are almost identical to the wild type stimulate immunologic memory cells and are considered potent vaccine candidates (35). Disruption of the activity of *Leishmania* genes could be achieved by knocking out one or two alleles. Parasites with one mutated allele, although showing a different phenotype from wild-type parasites, are considered dangerous vaccines due to the possibility of reversion. Knocking out two alleles results in loss of function (homozygous inactivation), thus maintaining survival in the host and culture environment and eliminating the risk of reactivation and pathogenesis, which could enhance immunity (25). The identification of *Leishmania* growth factors and virulence biomarkers, which play an important role in the immunomodulatory mechanisms and host interactions, was considered essential. The expansion of genetically live attenuated *Leishmania* vaccines could be improved through the attenuation of these biomarkers. Furthermore, the complete representation of the genetically live attenuated parasites prepares the analysis of the characteristics such as virulence and growth potential or the strength of immunogenicity (36).

There is strong evidence for the efficacy of genetically attenuated vaccines against malaria and leishmaniasis. Currently, mutant forms of Plasmodium falciparum have been produced that are reproducible parasites with the ability to be attenuated at the appropriate time of liver stage development, so-called early liver stage-arresting, replication-deficient (EARD) genetically attenuated parasites (GAP). These attenuated parasites were able to infect hepatocytes and transform into trophozoites (37). Next-generation GAPs, in addition to critical gene deletions, have acquired a specific gene sequence (gain of function) or additional function that results in the ability of the parasite to self-destruct at a desired time (38). Genetic knockout of the sporozoite liver-stage asparagine-rich protein (SLARP or SAP1) disrupts parasite growth in the primary liver stage before nuclear division. There is a broad consensus that the existence of the parasite in the hepatocyte, with its dynamic metabolism and restricted cell division, is necessary for long-term protection and immunity (39). The first in-human clinical trial and evaluation of the non-replicating, live, genetically attenuated *Plasmodium falciparum* sporozoite vaccine (PfSPZ-GA1), a double knockout parasite lacking the *b9* and *slarp* genes important for liver development (PfΔ*b*9Δ*slarp*), demonstrated safety, immunogenicity, and efficacy in malaria-naive Dutch volunteers (40).

In the case of genetically attenuated *Leishmania*, there is no limit to the selection of different target genes, provided that the disruption results in parasites that can infect cells and induce strong immunity without clinical observations. Various protein gene deletions such as metabolic enzymes, signaling pathway proteins, cell surface, and cytoskeleton-related proteins could be considered as suitable interventions (26) (Table 2). Namely, mutated *L. major* parasites with deletion of gene encoding the p27 protein (41), DHFR-TS (42, 43), GP63 (44), LPG (45), *Centrin1*, and many other genes have shown a significant reduction in parasite burden and symptoms as well as high immunity to challenge (46). Characterization of some live attenuated *L. donovani* vaccine candidates with deletion of the *Centrin1* and p27 genes has shown that the expression pattern of immunomodulatory proteins, such as HSP70 and tryparedoxin tubulins, DEAD-box RNA helicases, and host-protective proteins, including cytochrome c, calreticulin, and glyceraldehyde-3-phosphate dehydrogenase (GAPDH) are regulated in these parasites (47). Thus, these proteins could be studied as biomarkers for their role in attenuating the reproductive effect.

**Table 2:** Genetically engineered live attenuated Leishmania.

**Gene editing tool/gene**	**Function**	***Leishmania* strain**	**Animal/cell model**	**Consequence**	**References**
**Homologous recombination**					
Dihydrofolate reductase-thymidylate synthase (DHFR-TS)	DNA and pyrimidine synthesis	*Leishmania major*	BALB/c, Rhesus monkey	Low parasite burden and infection *in vivo* Potent immune response	(42) (43)
P27 protein	An element of cytochrome c oxidase complex associated with oxidative phosphorylation	*L. major*	Dogs	Indicating prolonged protection against virulent *Leishmania infantum* and no presence of lesion, reduced DTH reaction	(41)
Cysteine protease a and b (cpa/b)	An essential role in parasite pathogenesis	*Leishmania mexicana*	•BALB/c, C57BL/6, CBA/Ca •Hamster	Showed resistance, reduced parasite burden, and small lesions	(49) (50) (51)
B galactofuranosyl transferase (LPG 1)	Surface lipophosphoglycan synthesis	*L. major*	BALB/c	Showing a minimal delay in lesion induction	(45)
Sterol 24-c-methyltransferase (SMT)	Ergosterol synthesis	*L. major*	BALB/c, C57BL/6	Delayed in lesion induction and lower parasite load	(52)
Mannose-1-phosphate guanylyltransferase (GDP-MP)	Mannose donor in the glycosylation process	*L. mexicana*	BALB/c	Permanent immunity, complement susceptibility, decrease in parasite burden	(53)
2,4-dienoyl-coA reductase (DECR)	Essential for fatty acid β-oxidation	*L. major*	BALB/c	Reduced parasite burden	(54)
Alkyl-dihydroxy-acetonephosphate synthase (ADS1)	Ether lipid synthesis	*L. major*	BALB/c	Reduced parasite load, complement susceptibility	(55)
Fructose 1,6 bisphosphatase (FBP)	Essential role in gluconeogenesis	*L. major*	BALB/c	Induced protection against challenge, induced Th1 response, reduced parasite burden	(56)
Nucleobase transport (NT4)	Purine base uptake	*L. major*	BALB/c BMDM	Suppressing intracellular amastigotes	(57)
ATP-binding cassette protein subfamily G 1/2 (ABCG 1,2)	Membrane-bounded transporters responsible for drug resistance	*L. major*	BALB/c	•Low infection and parasite load •Homologous recombination	(58)
Mitochondrial carrier protein (MIT 1)	Iron transporter in mitochondria	*Leishmania amazonensis*	C57BL/6	No lesions, low parasite burden	(59)
Glucose transporter (GT) 1,2,3	Transport of glucose	*L. mexicana*	BALB/c	Low infection and parasite burden, without lesions	(60)
Kharon (KH)	Essential for flagellar transit of GT1, cytokinesis process and amastigote survival inside the cells	*L. mexicana*	BALB/c	Low parasite load, high IFN-γ, IgG, IL-17	(61)
Leishmanolysin (GP63)	Membranous metalloproteinase as an antigen involved in pathogenicity	*L. major*	BALB/c	Small lesions, complement, susceptibility.	(44)
KIN 29 DEATH kinesin	The motor protein inside the cell	*L. mexicana*	BALB/c	No appearance of lesion or disease	(62)
Bardet-bidle syndrome 1 protein-like (BBS 1)	Trafficking process related to primary cilium, in human	*L. major*	BALB/c	Low infection and parasite load, small lesions	(63)
Target of rapamycin kinase3 (TOR 3)	Regulation of cell proliferation and growth	*L. major*	BALB/c	Low parasite load and small lesions	(64)
PIWI-like protein 1 (PWI)	A mitochondrial argonate-like protein involved in the apoptosis process	*L. major*	BALB/c	Low parasite load and pathogenicity	(65)
Signal peptidase type 1 (SPase I)	Elimination of signal peptide portion of secretory proteins	*L. major*	BALB/c	Low parasite load, no lesion	(66)
**CRISPR-Cas system**					
*Centrin1* (*Cen 1*)	A cytoskeletal calcium-dependent protein involved in proliferation and centrosome duplication	•*L. mexicana* •*L. major*	BALB/c, C57BL/6 BMDM and BMDC	Increase in NO level, IFN-γ, IL-2, TNF-α and Th1 response. Decrease in anti-inflammatory cytokines and parasite load	(67) (48)
Eukaryotic translation initiation factor 4E-1 (eIF4E1)	Translation initiation factor	*L. mexicana*	RAW264.7 Macrophage	Low infection rate	(68)
Flagellum attachment zone protein 7 (FAZ 7)	Attachment of flagellum to the cell body involved in cytokinesis	*L. mexicana*	BALB/c	Low rate of growth and pathogenicity	(69)
Protein BTN1	Involved in vacuolar transport of Arg, also in Batten disease	*L. mexicana*	BALB/c	Parasite load and lesion size have no difference in WT and CRISPR groups	(70)
**diCre** ***loxP***					
Cdc2-related kinase 3 (*CRK3*)	Involved in *leishmania* proliferation, a functional homolog of CDK1	*L. mexicana*	BALB/c	Lower parasite burden and smaller lesion of the footpad	(71)

*L. major* mutant strains generated using advanced gene editing techniques, in which the targeted modification of the *Centrin* gene is accompanied by the insertion of an antibiotic resistance marker into the genome, are superior for development in Phase I human clinical trials. *L. major Centrin* gene-deleted parasites (*L.mCen*–/–) have also been shown to be safe and protective in immunodeficient mouse models. In addition, *LmCen*–/– parasites demonstrated immunity to sandfly challenge (48).

### Cre-*loxP* system

The Cre-*loxP* system has been used as a genetic engineering tool to enhance recombination between two *loxP* sequences for *in vivo/vitro* studies. The Cre recombinase gene is located near an inducible promoter to perform controllable or stage-specific gene deletion during the recombination process, which is advantageous for the phenotypic analysis of different genes. Genome editing by excision action of the Cre recombinase enzyme on the sequences flanked by the locus of crossover of the bacteriophage P1 (*loxP*) sites has been used in mammalian systems, given the absence of a regulated induction system, not long ago had not been administered to *Leishmania*. The advent of diCre technology overcame some of the system's drawbacks, such as sensitivity to leakage and promoter type. In this system, the Cre protein is cleaved into two functional inactive domains and lined to FKBP12 (FK506 binding protein) and FRB (binding domain of the FKBP12-rapamycin-associated protein). The addition of rapamycin or its analogs leads to fusion and activation of the separate domains, resulting in a recombination process between *loxP* sequences. This technique is an effective way to reduce the side effects of overexpression of active, potentially cytotoxic Cre recombinase. The diCre approach is unlikely to apply to some important genes that are organized in multi-copy arrays. Also, diCre will not avoid compensatory genetic reorganization in long-term null mutant studies (72, 73). For example, the inducible diCre system was used to knock out the *CRK3* gene in *Leishmania*, demonstrating the requirement for *CRK3* function in the regulation of mitosis and clearly showing growth failure in the cells 48 h after targeted deletion of *CRK3* (71).

### CRISPR

Clustered regulatory interspaced short palindromic repeats—the CRISPR/Cas system is a defense mechanism in bacterial microorganisms against foreign genetic material. CRISPR-Cas interference occurs when an infection occurs, and viruses or foreign plasmids enter the bacterial cell. After infection, unknown genetic sequences integrate into the bacterial CRISPR locus as spacer arrays, conferring immunity to subsequent infections associated with these viruses. RNA polymerase then transcribes pre-CRISPR RNAs (pre-crRNAs) from the spacer sequence of the CRISPR region, which eventually bind to Cas nucleases and form hydrogen bonds specifically with the DNA sequence target. This is accompanied by a transcription of the trans-activating crRNA (tracrRNA) from the CRISPR locus, leading to the maturation of the pre-crRNA by the enzyme RNase III and crRNA-directed DNA cleavage. The tracrRNA: crRNA complex is packaged with CRISPR-associated nuclease (Cas) to form a ribonucleoprotein (RNP) complex. This active complex releases Cas nuclease to create a double-strand break (DSB) in the DNA at the target sequence correlative to the crRNA sequence (72, 73). The Cas9 endonuclease, the class 2 type II CRISPR system, is the most widely used and precise genome editing tool. The first Cas9 endonuclease used in mammalian systems for gene editing belongs to *Streptococcus pyogenes*. The Cas9 enzyme has two endonuclease domains, RuvC and HNH, which cleaves the DNA strand non-complementary to the spacer sequence and the complementary strand, respectively (74, 75). Adhesion of the Cas-RNA complex to the target DNA spacer sequence (~20 nucleotides) near the protospacer adjacent motif (PAM 5′-NGG) induces the two Cas9 domains to cooperate, resulting in blunt double-strand breaks in DNA (76). Most of the DSBs could be repaired by DNA repair systems, including microhomology-mediated end joining (MMEJ) or homology direct repair (HDR) (77).

CRISPR technology has several advantages, such as its availability and simplicity for consumers, high efficiency, and suitability for genetic screening, which have allowed the application of this technique in all major fields (78). However, despite the efforts that have been made, there are some major concerns and limitations for the adoption of CRISPR/Cas9. The high incidence of off-target genome editing, probably more than 50%, has been observed and is mostly related to DNA modifications in non-specific regions or by misguidance of single guide RNA (sgRNA). An efficient approach to reduce off-target effects is to use Cas variants such as Cas9 nickase, which produces single-stranded breaks, whereas a double sgRNA targets both DNA strands at the target site and produces the DSB. Another limitation of CRISPR/Cas9 is the need for a PAM sequence adjacent to the target region.

CRISPR could cause DNA damage and apoptosis as a result of DSBs rather than the targeted gene editing (75). CRISPR has great superiority in indel efficiency in various cells compared to some gene-editing nucleases, but insufficient indels and high HDR could be increased depending on the variation of the target region (78). Designing an efficient gRNA for post-transcriptional modification of mRNA is a challenge for CRISPR technology. In 2014, Gao et al. designed an artificial gene RGR (ribozyme-gRNA-ribozyme) that promotes guide RNA production feasible (79). In addition, targeted delivery of CRISPR/Cas9 effectors is critical. Delivery methods vary depending on the cell type and include physical methods and viral methods (adenovirus or lentivirus vectors).

To date, major improvements in gene editing tools such as CRISPR technology have enabled the creation of genetically modified parasites with reduced virulence, persistent survival, and growth rate (35). Recent studies have shown that *Leishmania* strains, as polyploid organisms, have more than one set of chromosomes, and that genome evolution and repair mutations lead to the breakdown of the gene editing process. *Leishmania* could adapt to unstable situations through evolutionary mechanisms; furthermore, this parasite makes use of heterogeneous genome and regulatory procedures at different levels such as genomic, transcriptomic, and translational steps, which contribute to the ultimate survival and reversion of the pathogen so that genetic manipulation of crucial genes of trypanosomatids is considered more challenging than it seems (26, 80). Before the CRISPR-Cas9 era, gene deletion in *Leishmania* was more challenging due to low recombination capacity and the presence of an extra chromosome. Since the initial approval of CRISPR/Cas9 technology in *Trypanosoma cruzi, Leishmania*, and *Trypanosoma brucei*, gene replacement in trypanosomatids has become convenient and time-saving. It has also contributed to the study of basic biological mechanisms and functions in parasites (81).

**Figure 2:**
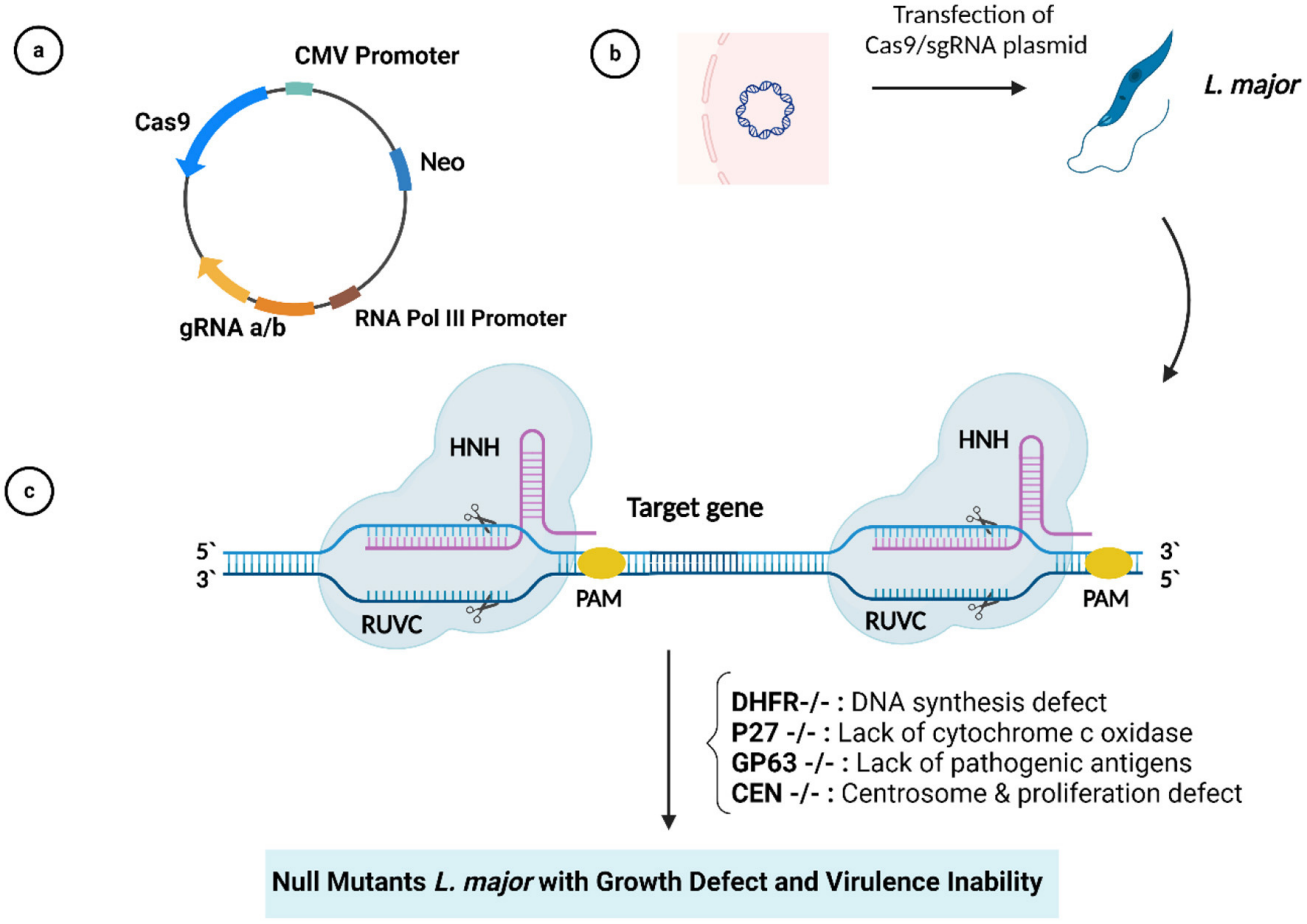
**(A)** Plasmid with Cas9/gRNA sequences. **(B)** Plasmid transfection into *Leishmania major*. **(C)** CRISPR/Cas9-mediated target gene deletion.

Second-generation leishmanization was presented by introducing an attenuated *L. major* strain mutated in the *Centrin1* gene (a cytoskeletal protein involved in mitosis) (*LmCen–/–*) using the CRISPR/Cas system (Figure 2). This attenuated parasite was found to be free of antibiotic resistance markers and there were no detectable off-target mutations, allowing it to be developed into a Phase 1 clinical trial. Animal models immunized with this attenuated vaccine showed a strong immune response but no visible lesions after the challenge with the infected sandfly, while non-immunized mice showed visible lesions and higher parasite loads. *LmCen–*/– is considered safe and effective compared to conventional leishmanization. It does not induce leishmaniasis in immunocompromised animals but does induce host immunity against sandfly infection (48). Of note, to fully exploit the editing potential of CRISPR/Cas9, they must be successfully delivered into target cells or tissues using appropriate viral and non-viral vectors, as reviewed in Goyal et al. (82) and Ayari-Riabi et al. (83).

Overall, new live vaccine platforms are also being explored but are still in the early stages of development for use against infectious pathogens. However, similar to classical whole-organism vaccine platforms, these novel vaccines also require the cultivation of the pathogen. Moreover, one of the disadvantages of this platform is that it must be delivered directly into cells, which requires a special injection device or a carrier molecule and carries the risk of low transfection rates and limited immunogenicity. However, next-generation live vaccines can be constructed using only the genetic sequence of the pathogen, significantly increasing the speed of development and manufacturing processes.

## Immunization of genetically live attenuated vaccines

**Figure 3:**
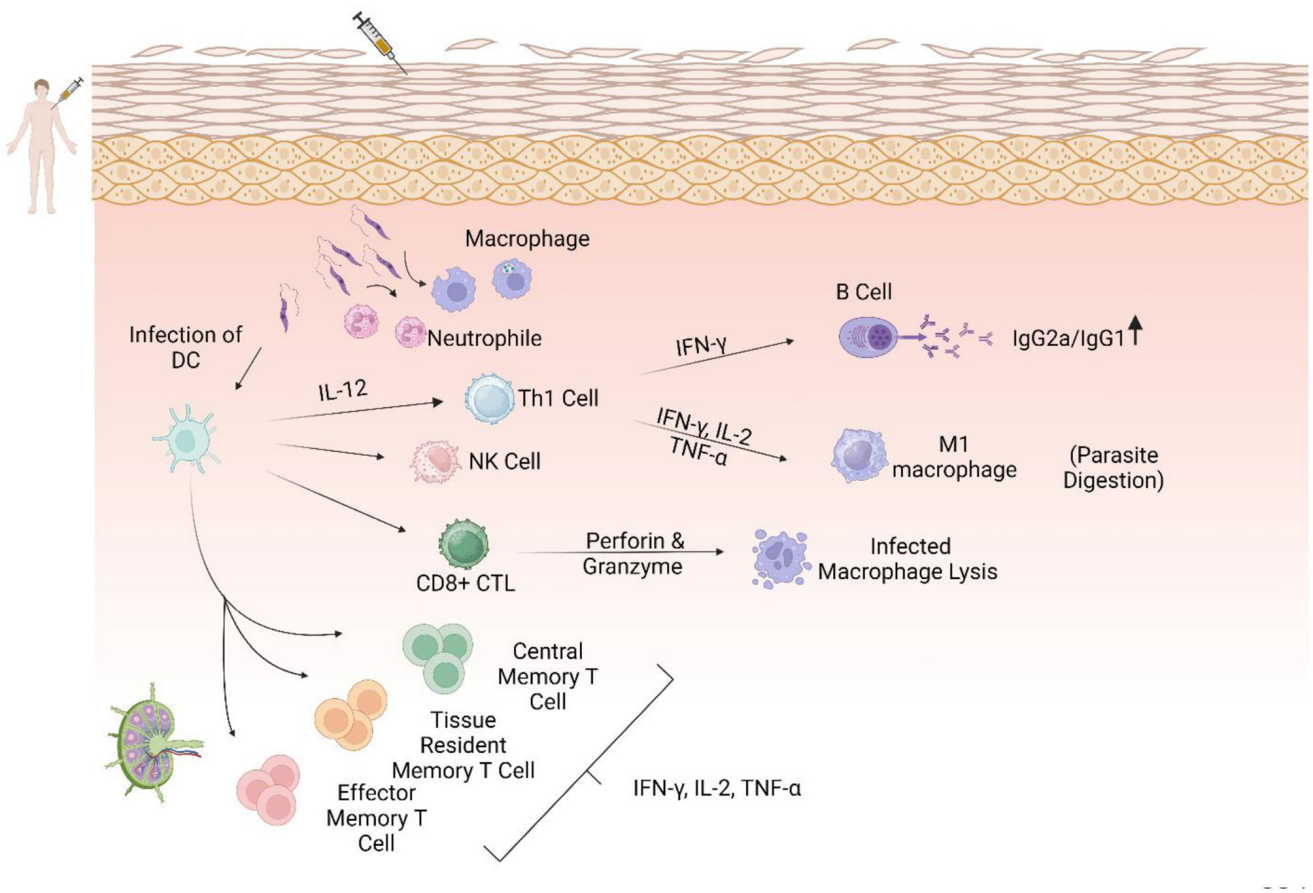
The central role of the Th1 response in immunization against leishmaniasis. Dendritic cells as antigen-presenting cells (APCs) after some interactions, including TLR4-L. LPG and phagocytic process, migrate to the lymphatic drains, activate CD4+ and CD8+ T cells, and secrete IL-12, which could promote a Th1 immune response. Th1 cells secrete pro-inflammatory cytokines such as IL-2, IFN-γ, and TNF-α, which activate M1 macrophages and increase NO and ROS production, leading to parasite clearance. In addition, CD8+ cells become cytotoxic T cells that produce perforin and granzymes that lyse infected macrophages. Increasing the ratio of IgG2a/IgG1 could lead to a protective immune response.

The development of genetically modified live attenuated *L. major Centrin*-deleted parasites as a second method of leishmanization could induce protection via the action of IFN-γ-secreting Ly6+CD4+ T effector cells and multifunctional T cells that secrete cytokines such as IFN-γ, which is necessary for their production and survival. The *LmCen–*/– vaccine could also generate CD4+ skin tissue-resident memory (TRM) T cells that proliferate at the site of infection and secrete more IFN-γ and granzyme B in immunized animal models (46, 48). Central memory T cells (TCM) and skin TRM have been characterized as *Leishmania*-independent memory T cells (Figure 3). TRM cells are particularly suitable for protection, probably due to their localization and recruitment following vaccination or *Leishmania* infection. Following the parasite challenge, TRM cells immediately begin to reduce parasite loads, and it has been suggested that development strategies involving these cells will be helpful in pursuit of a leishmaniasis vaccine (84).

In addition, Greta Volpedo et al. reported that immunization with *Centrin*-deficient *L. mexicana* also results in higher levels of IL-12 and generation of central memory T cells (CD4+CD44+CD62L+) and significantly higher Th1 immune responses in the skin and lymph nodes of BALB/c mice compared to non-immunized mice. Overall, the ratio of IFN-γ/IL-10 to IFN-γ/IL-4 represents the physiological balance between Th1 and Th2 responses that determines disease outcome and can make the difference between resistance and susceptibility. However, when compared to the New World *Leishmania* strains that cause cutaneous disease, *L. major* exhibits different immunological characteristics and pathologies. Analysis of metabolic responses in immune cells following immunization with *LmexCen–/–* revealed increased aspartate metabolism and pentose phosphate pathway (PPP), which induce M1 polarization in macrophages, and PPP also promotes nitric oxide production. In addition, increased taurine/hypotaurine metabolism at the site of infection and linoleic acid in lymph nodes could motivate macrophage and T-cell activation against the parasite. In addition, arachidonic acid (AA)—an endocannabinoid metabolite with significant anti-inflammatory properties—showed an escalation in the course of infection *in vivo*. In general, the discovery of metabolic and immunological interactions following *Leishmania* vaccination could improve the development of innovative strategies in vaccine formulation (67). Given the endemicity of CL, a vaccine that prevents severe disease could have a significant impact on public opinion. However, a live attenuated vaccine that could also block parasite infection and thus prevent both cutaneous manifestations would have a much greater impact by reducing community transmission and potentially establishing herd immunity. Advances in molecular parasitology, creating deleterious gene mutations, altering replication fidelity, optimizing codons, and exerting control through genetic engineering tools, particularly the CRISPR/Cas9 system, which offers new ways to control *L. major* infection and replication, are renewing interest in a new generation of live attenuated vaccines, although potentially safer and more broadly applicable live vaccines require further testing before further advancing to human trials.

## Conclusion

The spectrum of leishmaniasis varies due to host genetics and situation, parasite strain, and climate change. However, enough studies have shown that different forms of leishmaniasis can be prevented by vaccination. Unfortunately, there is currently no vaccine approved for human immunization on the global market. The development of an effective vaccine depends on its profitability for key stakeholders, vaccine developers, and manufacturers. Vaccine production requires a high level of trust in the public interest. Of course, government support attention to public health problems and international reflection are considered effective. Great advances have been made in the field of biological technologies to expand the range of vaccines. Recombinant multi-peptide adjuvanted vaccines such as *Leish*-F1 + MPL-SE and adenovirus-based DNA vaccines such as ChAd63-KH are now available. The priority of live attenuated *Leishmania* vaccines is considered to be a strong technique for the control of leishmaniasis, which has gained great attention due to the improvement of genetic engineering technologies such as the CRISPR/Cas system. The evaluation of gene candidates in terms of efficacy and immune response against the wild parasite has shown that *Centrin1* is the most encouraging and is recognized as a good option for genetically live attenuated *Leishmania* vaccines. As we know, all the *in vivo* studies have been performed in animal models, which represent the early stages of the development of genetically attenuated vaccines and have not yet reached human clinical trials. In general, confirmation of logical guidelines related to live attenuated *Leishmania* development could administer a fine direction to major studies before handling human clinical trials and seriously reorganize the timeline of vaccine candidates.

## Author contributions

ZR: Investigation, Visualization, Writing – original draft. HD-M: Conceptualization, Investigation, Project administration, Supervision, Validation, Visualization, Writing – review & editing. ME: Conceptualization, Investigation, Writing – review & editing. HK: Funding acquisition, Project administration, Resources, Supervision, Writing – review & editing.
